# The Association Between Sarcoidosis and Malignancy: A Comprehensive Population-Based Cohort Study

**DOI:** 10.3390/jcm13237045

**Published:** 2024-11-22

**Authors:** Yonatan Shneor Patt, Niv Ben-Shabat, Kassem Sharif, Chen Patt, Yoav Elizur, Mohamad Arow, Arnon D. Cohen, Abdulla Watad, Dennis McGonagle, Howard Amital, Paula David

**Affiliations:** 1 Department of Internal Medicine B, Sheba Medical Center, Tel-Hashomer, Ramat Gan 5262100, Israel; nivben7@gmail.com (N.B.-S.); kassemsharif@gmail.com (K.S.); chenktz@gmail.com (C.P.); yoav.elizur@mail.huji.ac.il (Y.E.); mohamad.arow@gmail.com (M.A.); watad.abdulla@gmail.com (A.W.); howard.amital@sheba.health.gov.il (H.A.); paulardavid@gmail.com (P.D.); 2 Faculty of Medicine, Tel-Aviv University, Tel-Aviv 6997801, Israel; 3 Department of Gastroenterology, Sheba Medical Center, Tel-Hashomer, Ramat Gan 5262100, Israel; 4 Chief Physician’s Office, Clalit Health Services, Tel-Aviv 6209813, Israel; arcohen@clalit.org.il; 5 Siaal Research Center for Family Medicine and Primary Care, Faculty of Health Sciences, Ben-Gurion University of the Negev, Beer-Sheva 8410501, Israel; 6 NIHR Leeds Musculoskeletal Biomedical Research Unit, Chapel Allerton, Leeds Teaching Hospital Trust, Leeds LS9 7TF, UK; d.g.mcgonagle@leeds.ac.uk; 7 Leeds Institute of Rheumatic and Musculoskeletal Medicine, University of Leeds, Leeds LS2 9JT, UK

**Keywords:** sarcoidosis, malignancy, cancer, lymphoma, epidemiology

## Abstract

**Background:** Sarcoidosis is a multisystem granulomatous disorder with a variable clinical course and complications. The relationship between sarcoidosis and malignancies remains unclear, including specific malignancy associations with sarcoidosis and whether the association is short-term, long-term, or a result of misdiagnoses or coincidence. This study investigated the association between sarcoidosis and malignancy by analyzing the varying intervals between the diagnoses of these two conditions to clarify their inter-relationship. **Methods:** This retrospective cohort study included almost 24,000 sarcoidosis patients and matched controls at a 1:5 ratio in patients diagnosed between 2000 and 2015 in Israel. Patients had a median age of 57 years. Malignancy rates were compared across several timeframes: overall, within one year before or after sarcoidosis diagnosis and more than one year. Logistic regression models were employed to estimate odds ratios for the association between sarcoidosis and malignancy, adjusting for sociodemographic and clinical variables. **Results:** Sarcoidosis patients had a significantly higher prevalence of malignancies (19.5%) compared to controls (13.6%) (*p* < 0.001). The association remained significant for both hematologic malignancies (OR: 2.94, 95% CI: 2.41–3.57) and solid malignancies (OR: 1.41, 95% CI: 1.27–1.55). The strongest association was observed with lymphoma, particularly within the first year of sarcoidosis diagnosis (OR: 14.88, 95% CI: 8.83–25.1). Elevated odds for malignancies persisted both within one year and beyond, including sarcoma and soft tissue cancers and genitourinary malignancies. **Conclusions:** Our study confirms a significant association between sarcoidosis and both hematologic and solid malignancies in both the short and long term across various timeframes. These findings emphasize the need for increased clinical vigilance in sarcoidosis patients and highlight the importance of further research into the shared genetic and environmental mechanisms that may underlie this relationship.

## 1. Introduction

Sarcoidosis is a multisystem granulomatous disorder of unknown etiology, characterized by non-caseating granulomas formation, most commonly the lungs and lymph nodes [1]. Sarcoidosis prevalence shows considerable global variation, with rates as low as 1–5 per 100,000 in regions like South Korea, Taiwan, and Japan, and as high as 140–160 per 100,000 in countries such as Sweden and Canada [2]. Risk factors include genetic predisposition, and environmental exposures, and possibly obesity, though the precise triggers remain unclear [2]. Infection has long been considered a potential risk factor for sarcoidosis due to its histological resemblance to other granulomatous diseases, like tuberculosis, and two case–control studies have demonstrated increased risk of sarcoidosis (odds in individuals with a prior infection history [3]. Diagnosis typically involves a combination of clinical evaluation, imaging (such as chest X-rays or CT scans), and histological confirmation of non-caseating granulomas, often obtained through biopsy [4]. Common symptoms include cough, shortness of breath, fatigue, and sometimes systemic symptoms like fever and weight loss [5]. The primary goals in sarcoidosis treatment are to reduce morbidity and mortality while improving quality of life, with lifestyle changes sometimes sufficient in mild cases, whereas drug treatment—including corticosteroids, methotrexate, and TNF-alpha inhibitors—is reserved for cases with significant organ involvement or symptoms that severely impact quality of life; however, these medications carry the risk of adverse effects and require careful management to avoid additional complications [5].

The clinical course of sarcoidosis varies significantly, with about half of all patients experiencing spontaneous resolution within two years of diagnosis, while others endure chronic and progressive symptoms that can result in substantial morbidity [6]. In some cases, sarcoidosis can lead to severe morbidity, including pulmonary fibrosis, an increased risk of ischemic heart disease, heart failure, and a poorer prognosis in pulmonary hypertension [7,8,9]. The potential relationship between sarcoidosis and malignancies was first proposed by Brincker in 1972, who suggested that patients with sarcoidosis might have an increased risk of malignancies [10]. Since then, numerous epidemiological studies reported inconsistent results [11,12]. In a comprehensive review, El Jammal et al. [12] summarized over 15 studies on this topic. While some studies found a clear association, others found no association, and a few identified links only with specific types of malignancies. In studies that report higher cancer rates, uncertainties persist regarding which malignancies—whether solid or hematologic—are more prevalent and whether the findings might reflect misdiagnoses, coincidence, paraneoplastic syndromes, or a true association between the two diseases [13]. The association between sarcoidosis and specific cancer sites, particularly lung cancer, has been widely debated and investigated in various studies. Two literature reviews on this topic concluded that, although the coexistence of sarcoidosis and lung cancer is rare, it remains a possibility. Awareness of these potential associations is crucial, as it may influence clinical management and guide appropriate disease monitoring [14,15].

This study seeks to extend previous longitudinal research by concurrently examining the association between sarcoidosis and malignancy across varying timelines, both before and after sarcoidosis diagnosis. Careful consideration of the time interval between the diagnoses of malignancy and sarcoidosis—whether they are made in close succession or over a longer duration—is crucial in clarifying the nature of the relationship between the two diseases. This approach allows for a more comprehensive assessment of the potential bidirectional relationship and helps differentiate between a genuine association and biases due to misdiagnosis or surveillance effects.

We leveraged a large-scale cohort from Israel’s largest Health Maintenance Organization (HMO) to investigate the association between sarcoidosis and malignancy in the Israeli population. By analyzing the varying intervals between the diagnoses of these two conditions, we aim to provide further understanding of their potential relationship in the context of existing literature, and to explore whether there is any genetic or shared pathological mechanism underlying this association.

## 2. Methods

### 2.1. Data Source

The dataset for this study was obtained from Clalit Healthcare Services (CHS), Israel’s largest health maintenance organization. CHS insures approximately 4.5 million members, representing a diverse population. The CHS database integrates patient data from pharmaceutical, medical, and administrative systems, enabling large-scale epidemiological research. Previous studies have validated the use of the CHS database for similar investigations [16,17].

### 2.2. Population and Study Design

In this retrospective study, we included all patients with an ICD-9 diagnosis code from hospital discharge letters or documentation from primary care or outpatient visits for sarcoidosis (ICD-9 135) recorded at least twice by two different physicians in the database between 1 January 2000 and 31 December 2015 to improve diagnostic accuracy. We did not exclude patients with a history of cancer, as our aim was to examine associations with cancer both before and after sarcoidosis diagnosis. All included patients were over the age of 18. For each sarcoidosis patient, we selected five age-, sex-, and residence-matched controls from the general population without a sarcoidosis diagnosis. The index date for each patient and their matched controls was defined as the date of the first recorded diagnosis of sarcoidosis.

### 2.3. Study Variables

Data collected included age (continuous), sex (categorical), socioeconomic status (SES) (categorical), obesity (categorical), smoking status (categorical), and comorbidities (categorical). SES was categorized using the poverty index from the 2008 National Census, which considered household income, education, marital status, and other socioeconomic indicators. Obesity was determined from body mass index (BMI) data (continuous) where available. Information on malignancies was obtained from the national cancer registry, with malignancies classified according to type and site.

### 2.4. Outcome

The primary outcome of this study was the diagnosis of malignancy, categorized by type and site. Malignancies were classified into two main groups: hematologic malignancies, which included leukemia (acute and chronic), Hodgkin’s lymphoma, Non-Hodgkin’s lymphoma, and multiple myeloma; and solid malignancies, which included gastrointestinal malignancies (esophageal, colorectal, stomach, liver and biliary, and pancreatic cancers), genitourinary malignancies (bladder, kidney, cervix, uterus, ovary, prostate, and genital cancers), and Ear nose and throat (ENT) malignancies (laryngeal, pharyngeal, and thyroid cancers). Other solid malignancies included were lung, breast, melanoma, sarcoma and soft tissue, central nervous system, and bone malignancies.

To better understand the nature of the association between sarcoidosis and malignancy, we assessed malignancy diagnoses across several timeframes. A short-term association, represented by a diagnosis of malignancy within one year before or after sarcoidosis diagnosis, may be influenced by factors such as misdiagnosis, increased medical surveillance due to frequent radiologic investigations, or sarcoidosis-like reactions. In contrast, a long-term association, defined as a malignancy diagnosis occurring more than one year before or after sarcoidosis diagnosis, is more likely to reflect a genuine association between the diseases. Therefore, we examined the following timeframes: overall, within one year before or after sarcoidosis diagnosis (or the index date for controls), and more than one year before or after sarcoidosis diagnosis (or the index date for controls).

### 2.5. Statistical Analysis

Baseline characteristics between sarcoidosis patients and controls were compared using *t*-tests or Mann–Whitney U tests for continuous variables, and Pearson’s χ^2^ tests for categorical variables. Logistic regression models were employed to estimate odds ratios (OR) and 95% confidence intervals (CI) for the association between sarcoidosis and malignancy, adjusting for potential confounders including age, sex, socioeconomic status, obesity, and smoking. The Benjamini–Hochberg method was applied to control for multiple comparisons. Subgroup analyses were performed based on the time from sarcoidosis diagnosis to malignancy diagnosis (within 1 year and more than 1 year). The results were presented as adjusted odds ratios with 95% CIs. All statistical analyses were conducted using SPSS for Windows (V.26.0, IBM SPSS Statistics, Armonk, NY, USA).

### 2.6. Ethics

The study was approved by the Clalit Healthcare Services Ethics Committee in Tel Aviv, Israel (approval number 0212-17-COM). Informed consent was not required due to the use of de-identified data.

## 3. Results

### 3.1. Study Population

A total of 3993 patients with sarcoidosis and 19,856 age-, sex-, and residence-matched controls were included in the study. The median age of both groups was 57.1 years, and females comprised 63.2% of the sarcoidosis group and 63.1% of the control group. SES was significantly different between the two groups (*p* < 0.001). A higher proportion of sarcoidosis patients were classified as having low SES (41.9%) compared to controls (37.7%), whereas the control group had a higher percentage of individuals with high SES (21.3% vs. 18.3% in the sarcoidosis group).

Regarding lifestyle factors, smoking rates were slightly lower in the sarcoidosis group (33.6%) compared to the controls (35.0%), though this difference did not reach statistical significance (*p* = 0.093). However, the prevalence of obesity was significantly higher among sarcoidosis patients (36.6%) than in the control group (28.9%) (*p* < 0.001) (Table 1).

### 3.2. Overall Malignancy Rates and Timing Relative to Sarcoidosis Diagnosis

Malignancies were significantly more common in sarcoidosis patients compared to controls. Overall, 19.5% of sarcoidosis patients were diagnosed with malignancies, compared to 13.6% of controls (*p* < 0.001). Hematologic malignancies occurred in 4.2% of sarcoidosis patients versus 1.5% of controls (*p* < 0.001), with specific differences noted for non-Hodgkin’s lymphoma (3.1% vs. 0.8%, *p* < 0.001) and Hodgkin’s lymphoma (0.8% vs. 0.3%, *p* < 0.001) as well as acute and chronic leukemia. 

Solid malignancies were also more frequent in sarcoidosis patients (20.7%) than in controls (12.5%) (*p* < 0.001). While most specific solid cancers did not differ significantly between the groups, several malignancies showed statistically significant differences, including sarcoma and soft tissue (0.6% vs. 0.2%, *p* < 0.001), kidney (0.8% vs. 0.5%, *p* = 0.014), lung cancer (1.3% vs. 0.8%, *p* = 0.008) and unknown primary (2.9% vs. 1.1%, *p* < 0.001).

When analyzed in relation to the timing of sarcoidosis diagnosis, 11.0% of sarcoidosis patients were diagnosed with malignancies before their sarcoidosis diagnosis, compared to 7.5% of controls (*p* < 0.001). Hematologic malignancies were more common in sarcoidosis patients before diagnosis (1.9% vs. 0.8% in controls, *p* < 0.001), with significant differences observed in the rates of non-Hodgkin’s lymphoma (1.3% vs. 0.4%, *p* < 0.001) and Hodgkin’s lymphoma (0.5 vs. 0.2 in controls, *p* < 0.001). Solid malignancies were also more frequent before sarcoidosis diagnosis (9.4% in sarcoidosis patients vs. 6.8% in controls, *p* < 0.001). Among specific solid malignancies, significant differences were noted for cervix (0.3% vs. 0.1%, *p* = 0.014), sarcoma and soft tissue (0.3% vs. 0.1%, *p* = 0.01), kidney (0.5% vs. 0.2%, *p* = 0.026), lung (0.5% vs. 0.2%, *p* = 0.018), uterine cancer (0.6% vs. 0.3%, *p* = 0.003) and unknown primary (1.6% vs. 0.7%, *p* < 0.001).

After sarcoidosis diagnosis, malignancy rates remained elevated, with 9.2% of sarcoidosis patients developing malignancies compared to 6.7% of controls (*p* < 0.001). Hematologic malignancies continued to be significantly more common in sarcoidosis patients post-diagnosis (2.2% vs. 0.8%, *p* < 0.001), particularly Hodgkin’s lymphoma (0.3% vs. 0.1%, *p* = 0.003), non-Hodgkin’s lymphoma (1.5% vs. 0.4%, *p* < 0.001) and acute leukemia (0.4% vs. 0.2%, *p* = 0.005). The incidence of solid malignancies also remained higher in sarcoidosis patients (7.4% vs. 6.1%, *p* = 0.002). Among specific solid malignancies, lung and kidney cancers, which were significantly elevated before the sarcoidosis diagnosis, no longer showed a significant difference after diagnosis. However, sarcoma and soft tissue cancers and unknown primary continued to show a higher incidence in sarcoidosis patients even after diagnosis (0.4% vs. 0.1%, *p* < 0.001; 1.2% vs. 0.4%, *p* < 0.001, respectively). For further details, see Table 2.

### 3.3. Malignancy Rates Within One Year and Beyond Sarcoidosis Diagnosis

When analyzing malignancies within one year before or after sarcoidosis diagnosis, malignancies were significantly more frequent in sarcoidosis patients (5.5%) compared to controls (1.9%) (*p* < 0.001). Hematologic malignancies were particularly elevated in sarcoidosis patients (1.8% vs. 0.2%, *p* < 0.001), with notable differences in non-Hodgkin’s lymphoma (1.4% vs. 0.1%, *p* < 0.001), Hodgkin’s lymphoma (0.3% vs. 0.0%, *p* < 0.001), acute leukemia (0.2% vs. 0.0%, *p* < 0.001) and multiple myeloma (0.2% vs. 0.0%, *p* < 0.001). Solid malignancies were also more common in sarcoidosis patients during this period (3.8% vs. 1.8%, *p* < 0.001), with significant differences noted for sarcoma and soft tissue (0.2% vs. 0.0%, *p* = 0.002), kidney (0.3% vs. 0.1%, *p* < 0.001), lung (0.5% vs. 0.1%, *p* < 0.001), pancreas (0.2% vs. 0.0%, *p* = 0.004), thyroid (0.1% vs. 0.0%, *p* = 0.021) and unknown primary malignancies (0.8% vs. 0.1%, *p* < 0.001).

In the period extending beyond one year before and after sarcoidosis diagnosis, malignancy rates remained significantly elevated in sarcoidosis patients (14.0%) compared to controls (11.7%) (*p* < 0.001). Hematologic malignancies continued to be more frequent in sarcoidosis patients (2.4% vs. 1.3%, *p* < 0.001), with substantial differences observed for both Hodgkin’s lymphoma (0.5% vs. 0.2%, *p* = 0.006) and non-Hodgkin’s lymphoma (1.7% vs. 0.7%, *p* < 0.001). Solid malignancies also remained more common in sarcoidosis patients (12.4% vs. 10.7%, *p* = 0.002). However, only sarcoma and soft tissue malignancies (0.5% vs. 0.2%, *p* < 0.001) and unknown primary malignancies (2.1% vs. 1.0%, *p* < 0.001) showed a significant difference. For further details, see Table 3.

### 3.4. Multivariate Analysis of Malignancy Odds in Sarcoidosis Patients Compared to Controls

The multivariate logistic regression analysis demonstrated that sarcoidosis patients had significantly higher odds of developing malignancies compared to controls, both overall and within specific timeframes. Overall, sarcoidosis patients had 1.61 times higher odds of developing malignancies compared to controls (95% CI: 1.47–1.76, *p* < 0.001). The odds were elevated for both hematologic malignancies (OR: 2.94, 95% CI: 2.41–3.57, *p* < 0.001) and solid malignancies (OR: 1.41, 95% CI: 1.27–1.55, *p* < 0.001). Among specific malignancies, sarcoidosis patients had significantly higher odds for lymphoma (OR: 3.67, 95% CI: 2.92–4.63, *p* < 0.001), leukemia (OR: 1.97, 95% CI: 1.28–3.04, *p* = 0.002), lung cancer (OR: 1.59, 95% CI: 1.15–2.19, *p* = 0.005), genitourinary malignancies (OR: 1.32, 95% CI: 1.10–1.59, *p* = 0.003), and sarcoma and soft tissue malignancies (OR: 2.71, 95% CI: 1.61–4.58, *p* < 0.001).

Within one year of sarcoidosis diagnosis, the odds of developing malignancies were even higher, with an adjusted OR of 3.04 (95% CI: 2.56–3.61, *p* < 0.001) compared to controls. The largest increase was seen in hematologic malignancies, with sarcoidosis patients having 11.37 times higher odds (95% CI: 7.43–17.4, *p* < 0.001) of developing these cancers, particularly lymphoma (OR: 14.88, 95% CI: 8.83–25.1, *p* < 0.001). Solid malignancies also remained significantly elevated (OR: 2.24, 95% CI: 1.83–2.72, *p* < 0.001), particularly lung cancer (OR: 4.49, 95% CI: 2.44–8.26, *p* < 0.001), genitourinary malignancies (OR: 1.93, 95% CI: 1.29–2.90, *p* = 0.001), and sarcoma and soft tissue malignancies (OR: 3.42, 95% CI: 1.18–9.91, *p* = 0.024).

For more than one year after sarcoidosis diagnosis, the odds of developing malignancies remained significantly elevated in sarcoidosis patients (OR: 1.26, 95% CI: 1.14–1.40, *p* < 0.001). Hematologic malignancies had higher odds (OR: 1.83, 95% CI: 1.44–2.33, *p* < 0.001), and solid malignancies also remained elevated (OR: 1.21, 95% CI: 1.09–1.35, *p* < 0.001). Among specific malignancies, sarcoidosis patients had higher odds of developing lymphoma (OR: 2.23, 95% CI: 1.68–2.96, *p* < 0.001), while the odds for leukemia were not significantly elevated. For solid malignancies, only sarcoma and soft tissue malignancies continued to show significantly higher odds in sarcoidosis patients (OR: 2.52, 95% CI: 1.38–4.61, *p* = 0.003). Genitourinary malignancies also showed slightly higher odds in sarcoidosis patients (OR: 1.23, 95% CI: 1.00–1.50, *p* = 0.047). For further details, see Table 4.

## 4. Discussion

This study demonstrated a significantly higher prevalence of malignancies among sarcoidosis patients compared to controls (19.5% vs. 13.6%, *p* < 0.001). This association remained robust even after adjusting for potential confounders such as age, sex, socioeconomic status, obesity, and smoking (OR: 1.61, 95% CI: 1.47–1.76). Notably, the association encompassed both hematologic and solid malignancies, occurring across various timeframes—before and after the diagnosis of sarcoidosis, and within the one-year and beyond-one-year periods relative to sarcoidosis diagnosis. The association was particularly prominent for lymphoma (which reached an OR of 14.88), and the strongest relationship was observed within the first year before and after the sarcoidosis diagnosis.

Our finding that the OR for hematologic malignancies, particularly lymphoma, was the most significant, aligns well with early [10,18] and recent studies [19]. Indeed, the association was primarily observed with lymphoma, leading to the coining of the term “sarcoidosis-lymphoma syndrome”. Several hypotheses have been proposed to explain this pronounced association with hematologic malignancies. One possible explanation is the increased mitotic activity of lymphocytes in sarcoidosis due to the immune response, which could raise the likelihood of mutations and subsequent malignant transformation [20]. Regarding the reverse relationship, the unique association seen among lymphoma patients with sarcoidosis may be due to lymphoma’s tendency to involve intrathoracic lymph nodes, potentially triggering sarcoidosis more readily than extrathoracic tumors [21]. Additionally, the widespread use of immune checkpoint inhibitors (ICIs) in hematologic patients could contribute to this association, as ICIs are known to cause several immune-related adverse events, including ICI-induced sarcoidosis-like reactions [22,23].

In addition to the observed association with hematologic malignancies, our study also demonstrated a positive association between sarcoidosis and solid malignancies across several time periods. The specific solid malignancies that showed statistically significant associations were sarcoma and soft tissue cancers, genitourinary cancers, and lung cancers, both overall and within one year of sarcoidosis diagnosis. However, lung cancer did not maintain statistical significance beyond one year before and after the diagnosis of sarcoidosis. Previous studies have suggested an increased association between sarcoidosis and malignancies affecting organs that are frequently targeted by the inflammatory process of sarcoidosis, particularly the kidney and skin [19,24]. 

Nonetheless, several studies have been unable to demonstrate a consistent long-term association between lung cancer and sarcoidosis, even though inflammation of lung tissue could theoretically serve as a risk factor for carcinogenesis [19,24,25]. One plausible explanation for the reduced long-term association between lung cancer and sarcoidosis may be related to the higher prevalence of nonsmokers among sarcoidosis patients, a phenomenon also observed in our study (albeit without reaching statistical significance) [26]. This could be attributed to the effect of smoking cessation; it often takes several years for the risk of lung cancer to decrease after a patient quits smoking. Another possible explanation for the diminished association between lung cancer and sarcoidosis over time could be the poor prognosis and reduced life expectancy associated with lung cancer, which may limit the detection of this association.

Interestingly, our study also revealed a significant increase in the association with sarcoma and soft tissue cancers, a finding that has not been reported in previous literature. One possible explanation could be the proximity of soft tissues to the skin, which is a known site frequently affected by sarcoidosis. Unfortunately, our study did not include data on non-melanoma skin cancers, which limits our ability to explore this potential connection further. Additionally, sarcoma is a rare disease, so some of these cases might actually represent cutaneous or infiltrating granulomatous lesions that were misdiagnosed as sarcoma.

The short-term association between sarcoidosis and malignancy, particularly in the form of paraneoplastic sarcoidosis and sarcoidosis-like reactions (SLR), has been well recognized for several years [18]. This phenomenon involves the presentation of granulomatous reactions, often found in the draining lymph nodes of a tumor, which do not fulfill the criteria for systemic sarcoidosis and typically occurs shortly before or after the detection of sarcoidosis [27]. Notably, this short-term association could be attributed to other factors such as misdiagnoses or increased medical surveillance bias due to more frequent radiologic investigations in sarcoidosis patients, all of which are more likely to occur during the initial year following the diagnosis of sarcoidosis or malignancy. Nevertheless, beyond the short-term association, numerous epidemiological studies have been conducted to investigate whether a true association exists between systemic sarcoidosis and malignancy in the long term, but the results have been unclear [28]. For example, a large Danish nationwide cohort study of 12,890 patients with a first-time sarcoidosis diagnosis found an elevated risk of malignancy across several timeframes, including 0–3 months, 3 months–3 years, 3–10 years, and beyond 10 years. In contrast, a meta-analysis by Ungprasert et al. [4]. reported a pooled RR of 1.21 for malignancy in sarcoidosis patients, but this association lost significance when cases within the first year after diagnosis were excluded. 

Interestingly, the reverse relationship, in which malignancy precedes sarcoidosis, beyond the short-term SLR or paraneoplastic association, has also been reported in several studies [29,30,31,32]. However, the specific literature on this phenomenon is limited, consisting primarily of case series without the support of large cohort studies [24,33]. In a series of 29 sarcoidosis patients with pre-existing cancer, Arish et al. [21] concluded that the association between malignancy and sarcoidosis was indicative of genuine systemic sarcoidosis rather than a localized SLR. 

Given the potential bidirectional relationship between sarcoidosis and malignancy, our study is the first to concurrently explore and confirm a clear association between these two conditions both before and after the diagnosis of sarcoidosis. This approach also addresses the inherent difficulty in determining the chronological sequence of the two diseases, as what initially appears to be a diagnosis of sarcoidosis may, in fact, be an SLR representing an underlying primary malignancy that has not yet been detected [34].

The observed association between sarcoidosis and malignancy may be explained by shared genetic and environmental factors that contribute to a common pathway of immunological dysregulation, predisposing individuals to both conditions. Immunologic abnormalities in sarcoidosis, coupled with chronic, uncontrolled inflammation, could impair tumor cell rejection [20] and lead to the development of specific cytokines that create a pro-inflammatory tumor microenvironment [24,35,36]. Additionally, malignancies, whether influenced by chemotherapy or not, may contribute to immune dysfunction that prevents the host’s phagocytic cells from clearing inciting agents or immune complexes. This impairment could then trigger an abnormal immune response, leading to the formation of granulomas and the subsequent development of sarcoidosis or SLR [37]. 

A notable environmental factor potentially contributing to the association between sarcoidosis and malignancy is obesity. Obesity has been previously associated with an elevated risk of developing sarcoidosis, which could partly explain the higher prevalence of obesity observed in our sarcoidosis cohort compared to matched controls [38,39]. Obesity is also a well-established predisposing factor for numerous malignancies [40]. The secretion of adipokines by adipose tissue contributes to a pro-inflammatory environment characterized by chronic inflammation and altered cytokine levels. This chronic inflammatory state not only plays a role in sarcoidosis development but also creates oxidative stress, which can damage DNA and increase susceptibility to cancer [41]. However, the association remained strong also after adjusting for obesity.

While this study offers valuable and robust conclusions, including both in-patient and outpatient cases, certain limitations arise from the use of medical registry-based databases. In our cohort, the diagnosis of sarcoidosis was based on ICD-9 coding, which represented sarcoidosis with a single code (ICD-9 Code: 135) without further classification into specific organ involvement. This limited our ability to explore correlations between cancer risk and the specific localization or manifestation of sarcoidosis as described in ICD-11. Similarly, the staging of oncologic diseases at the time of diagnosis was not available, restricting our ability to examine whether sarcoidosis patients are more likely to present with advanced or early-stage malignancies. Additionally, biopsy confirmation results, which are considered the gold standard for sarcoidosis diagnosis, were not accessible. Also, other detailed clinical information, such as disease severity and other individualized patient characteristics, was not accessible. However, a previous study examining the chest X-ray patterns of sarcoidosis found no evidence that disease severity predicts the risk of malignancy [42]. 

An additional limitation lies in the scope of the present analysis, which did not extend to malignancy-related mortality among sarcoidosis patients, which could have provided valuable insights. This specific association was not addressed in this manuscript because the primary focus was on investigating the occurrence of cancer in relation to sarcoidosis, both before and after diagnosis. Analyzing mortality would require a different study design and methodological approach, rather than the odds ratios employed in this analysis. Another limitation is the lack of data on the specific therapeutic agents received by the patients. Immunosuppressive agents could potentially increase the rates of certain cancers, especially hematologic and skin cancers. Nonetheless, it should be noted that only a minority of sarcoidosis patients, primarily those with steroid-refractory cases, are treated with immunosuppressive agents rather than corticosteroids. Therefore, it is unlikely that this potential bias significantly impacted our overall results [24,43].

## 5. Conclusions

Our study demonstrates both the short- and long-term associations between sarcoidosis and malignancy, encompassing both solid and hematologic malignancies. These findings underscore the importance of maintaining a high level of clinical vigilance in patients with sarcoidosis, as there may be an increased likelihood of developing a malignancy—and vice versa. Any clinical or imaging changes should prompt physicians to reassess and consider biopsy to exclude other diagnoses.

Future research should explore correlations between cancer risk and the specific localization or manifestation of sarcoidosis, utilizing more detailed classification systems such as ICD-11. Furthermore, investigations into malignancy-related mortality and relapse-free survival among sarcoidosis patients are needed. From a pathophysiological perspective, understanding the shared genetic and environmental factors contributing to the development of both conditions may provide valuable insights into the mechanisms underlying their association, potentially influencing research priorities and clinical practice.

## Figures and Tables

**Table 1:** Baseline characteristics of the study population.

Characteristics	Sarcoidosis (*n* = 3993)	Controls (*n* = 19,856)	*p*-Value
Age, median (IQR)	57.1 (46.1–67.1)	57.1 (46.0–67.0)	0.872
Female gender	2522 (63.2%)	12,527 (63.1%)	0.932
socioeconomic status			<0.001
Low	1654 (41.9%)	7376 (37.7%)	
Intermediate	1569 (39.8%)	8029 (41.0%)	
High	723 (18.3%)	4168 (21.3%)	
Smoking	1342 (33.6%)	6494 (35.0%)	0.093
Obesity	1462 (36.6%)	5736 (28.9%)	<0.001

**Table 2:** Rates of malignancies compared between sarcoidosis patients and controls; overall, before and after sarcoidosis diagnosis/index date for controls.

Malignancy Type/Site	Time Difference Between Sarcoidosis to Malignancy; Months; Median (IQR)	Overall			Before Sarcoidosis Diagnosis/Index Date			After Sarcoidosis Diagnosis/Index Date		
		Malignancy Rate in Sarcoidosis Patients; *n*(%)	Malignancy Rate in Controls; *n*(%)	*p*-Value	Malignancy Rate in Sarcoidosis Patients; *n*(%)	Malignancy Rate in Controls; *n*(%)	*p*-Value	Malignancy Rate in Sarcoidosis Patients; *n*(%)	Malignancy Rate in Controls; *n*(%)	*p*-Value
All Malignancies	38 (10–87)	779 (19.5)	2696 (13.6)	**<0.001 ^a^**	439 (11.0)	1488 (7.5)	**<0.001 ^a^**	367 (9.2)	1323 (6.7)	**<0.001 ^a^**
Hematologic	19 (3–53)	168 (4.2)	292 (1.5)	**<0.001 ^a^**	75 (1.9)	151 (0.8)	**<0.001 ^a^**	87 (2.2)	149 (0.8)	**<0.001 ^a^**
Acute Leukemia	44 (7–87)	22 (0.6)	54 (0.3)	**0.004 ^a^**	6 (0.2)	19 (0.1)	0.331	16 (0.4)	35 (0.2)	**0.005 ^a^**
Chronic Leukemia	45 (29–70)	14 (0.4)	36 (0.2	**0.033**	8 (0.2)	19 (0.1)	0.073	6 (0.2)	17 (0.1)	0.230
Hodgkin’s Lymphoma	16.3 (4–59)	33 (0.8)	55 (0.3)	**<0.001 ^a^**	20 (0.5)	34 (0.2)	**<0.001 ^a^**	12 (0.3)	21 (0.1)	**0.003 ^a^**
Non-Hodgkin’s Lymphoma	19 (2–61)	123 (3.1)	161 (0.8)	**<0.001 ^a^**	52 (1.3)	83 (0.4)	**<0.001 ^a^**	59 (1.5)	78 (0.4)	**<0.001 ^a^**
Multiple Myeloma	14 (2–35)	14 (0.4)	47 (0.2)	0.193	8 (0.2)	25 (0.1)	0.248	6 (0.2)	22 (0.1)	0.506
Solid	44 (13–95)	646 (20.7)	2474 (12.5)	**<0.001 ^a^**	375 (9.4)	1360 (6.8)	**<0.001 ^a^**	294 (7.4)	1204 (6.1)	**0.002 ^a^**
Breast	68 (34–132)	157 (3.9)	718 (3.6)	0.333	107 (2.7)	467 (2.4)	0.218	50 (1.3)	251 (1.3)	0.951
Bladder	54 (4–90)	23 (0.6)	143 (0.7)	0.317	11 (0.3)	59 (0.3)	0.817	12 (0.3)	84 (0.4)	0.265
Bone	-	0	13 (0.1)	0.106	0	4(0.0)	0.370	0	9 (0.0)	0.178
Central nervous system	28 (16–90)	13 (0.3)	34 (0.2)	0.045	4 (0.1)	11 (0.1)	0.303	8 (0.2)	23 (0.1)	0.176
Cervix	43 (17–80)	14 (0.4)	43 (0.2)	0.113	12 (0.3)	26 (0.1)	**0.014 ^a^**	1 (0.0)	17 (0.1)	0.203
Colorectal	71 (33–113)	74 (1.9)	355 (1.8)	0.777	42 (1.1)	176 (0.9)	0.316	31 (0.8)	179 (0.9)	0.440
Sarcoma and soft tissue	34 (8–38)	25 (0.6)	41 (0.2)	**<0.001 ^a^**	10 (0.3)	19 (0.1)	**0.010 ^a^**	14 (0.4)	22 (0.1)	**<0.001 ^a^**
Esophagus	-	3 (0.1)	17 (0.1)	0.835	0 (0)	2(0)	0.526	3 (0.1)	15 (0.1)	0.993
Kidney	37 (7–92)	33 (0.8)	101 (0.5)	**0.014 ^a^**	18 (0.5)	49 (0.2)	**0.026**	15 (0.4)	52 (0.3)	0.215
Larynx	79 (39–104)	7 (0.2)	32 (0.2)	0.840	3 (0.1)	18 (0.1)	0.763	4 (0.1)	14 (0.1)	0.533
Liver and Bile	26 (7–60)	11 (0.3)	38 (0.2)	0.284	3 (0.1)	9 (0.443)	0.443	8 (0.2)	29 (0.1)	0.426
Lung	26 (1–61)	50 (1.3)	163 (0.8)	**0.008 ^a^**	18 (0.5)	47 (0.2)	**0.018**	30 (0.8)	116 (0.6)	0.217
Genital	20 (6–49)	8 (0.2)	24 (0.1)	0.211	5 (0.1)	12 (0.1)	0.162	3 (0.1)	12 (0.1)	0.735
Ovary	58 (22–113)	16 (0.4)	50 (0.3)	0.102	7 (0.2)	27 (0.1)	0.548	9 (0.2)	23 (0.1)	0.084
Pancreas	30 (8–114)	19 (0.5)	74 (0.4)	0.340	5 (0.1)	13 (0.1)	0.210	14 (0.4)	61 (0.3)	0.655
Pharynx	14 (5–93)	9 (0.2)	50 (0.3)	0.759	5 (0.1)	23 (0.1)	0.874	3 (0.1)	27 (0.1)	0.322
Prostate	41 (13–97)	43 (1.1)	161 (0.8)	0.096	17 (0.4)	85 (0.4)	0.984	26 (0.7)	76 (0.4)	**0.018**
Thyroid	32 (9–76)	28 (0.7)	100 (0.5)	0.119	18 (0.5)	68 (0.3)	0.297	10 (0.3)	32 (0.2)	0.220
Uterus	76 (31–104)	32 (0.8)	122 (0.6)	0.178	23 (0.6)	55 (0.3)	**0.003 ^a^**	9 (0.2)	67 (0.3)	0.252
Melanoma	54 (14–151)	30 (0.8)	165 (0.8)	0.610	20 (0.5)	99 (0.5)	0.985	10 (0.3)	66 (0.3)	0.402
Stomach	39 (19–79)	8 (0.2)	64 (0.3)	0.200	1 (0.0)	28 (0.1)	0.055	7 (0.2)	36 (0.2)	0.935
Another site	32 (4–82)	35 (0.9)	115 (0.6)	**0.030**	15 (0.4)	49 (0.2)	0.151	16 (0.4)	66 (0.3)	0.501
Unknown primary	32 (9–76)	115 (2.9)	216 (1.1)	**<0.001 ^a^**	65 (1.6)	131 (0.7)	**<0.001 ^a^**	46 (1.2)	85 (0.4)	**<0.001 ^a^**

**^a^** statistically significant at a level of *p* < 0.05 after accounting for multiple comparisons using the Benjamini–Hochberg method. Comparison of malignancy rates and time between malignancy and sarcoidosis diagnosis/index date in sarcoidosis patients versus matched controls. Data is presented as the median time (in months) from sarcoidosis diagnosis to malignancy onset or the opposite, with interquartile range (IQR) shown in parentheses. Malignancy rates are given as the number of cases (*n*) and percentage (%) within each group. *p*-values indicate statistical significance for comparisons between sarcoidosis patients and controls. Results are divided into overall malignancies, malignancies diagnosed before sarcoidosis, and malignancies diagnosed after sarcoidosis. The “All Malignancies” category encompasses both hematologic and solid tumors, while specific malignancy types/sites are listed individually. Bold *p*-values (indicated by “a”) denote statistical significance (*p* < 0.05).

**Table 3:** Rates of malignancies compared between sarcoidosis patients and controls in a period of 1 year before diagnosis/index date versus longer than 1 year.

Malignancy Type/Site	Within 1 Year Difference Between Diagnosis of Sarcoidosis/Index Date to Diagnosis of Malignancy			More than 1 Year Difference Between Diagnosis of Sarcoidosis/Index Date to Diagnosis of Malignancy		
	Malignancy Rate in Sarcoidosis Patients; *n*(%)	Malignancy Rate in Controls; *n*(%)	*p*-Value	Malignancy Rate in Sarcoidosis Patients; *n*(%)	Malignancy Rate in Controls; *n*(%)	*p*-Value
All Malignancies	218 (5.5)	379 (1.9)	<0.001 ^a^	561 (14.0)	2317 (11.7)	<0.001 ^a^
Hematologic	72 (1.8)	33 (0.2)	<0.001 ^a^	96 (2.4)	259 (1.3)	<0.001 ^a^
Acute Leukemia	7 (0.2)	6 (0.0)	<0.001 ^a^	15 (0.4)	48 (0.2)	0.133
Chronic Leukemia	2 (0.1)	3 (0.0)	0.164	12 (0.3)	33 (0.3)	0.074
Hodgkin’s Lymphoma	13 (0.3)	6 (0.0)	<0.001 ^a^	20 (0.5)	49 (0.2)	0.006 ^a^
Non-Hodgkin’s Lymphoma	54 (1.4)	19 (0.1)	<0.001 ^a^	69 (1.7)	142 (0.7)	<0.001 ^a^
Multiple Myeloma	7 (0.2)	5 (0.0)	<0.001 ^a^	7 (0.2)	42 (0.2)	0.645
Solid	150 (3.8)	349 (1.8)	<0.001 ^a^	496 (12.4)	2125 (10.7)	0.002 ^a^
Breast	15 (0.4)	87 (0.4)	0.581	142 (3.6)	631 (3.2)	0.218
Bladder	7 (0.2)	20 (0.1)	0.201	16 (0.4)	123 (0.6)	0.098
Bone	0	1 (0.0)	0.654	0	12 (0.1)	0.120
Central nervous system	2 (0.1)	3 (0.0)	0.164	11 (0.3)	31 (0.2)	0.101
Cervix	2 (0.1)	5 (0.0)	0.402	12 (0.3)	38 (0.2)	0.169
Colorectal	7 (0.2)	49 (0.2)	0.395	67 (1.7)	306 (1.5)	0.525
Sarcoma and soft tissue	7 (0.2)	8 (0.0)	0.002 ^a^	18 (0.5)	33 (0.2)	<0.001 ^a^
Esophagus	0 (0)	2 (0.0)	0.526	3 (0.1)	15 (0.1)	0.993
Kidney	13 (0.3)	14 (0.1)	<0.001 ^a^	20 (0.5)	87 (0.4)	0.588
Larynx	0 (0)	3 (0.0)	0.437	7 (0.2)	29 (0.1)	0.664
Liver and Bile	3 (0.1)	9 (0.0)	0.443	8 (0.2)	29 (0.1)	0.426
Lung	20 (0.5)	22 (0.1)	<0.001 ^a^	30 (0.8)	141 (0.7)	0.778
Genital	3 (0.1)	6 (0.0)	0.182	5 (0.1)	18 (0.1)	0.521
Ovary	3 (0.1)	7 (0.0)	0.261	13 (0.3)	43 (0.2)	0.194
Pancreas	7 (0.2)	9 (0.0)	0.004 ^a^	12 (0.3)	65 (0.3)	0.785
Pharynx	4 (0.1)	13 (0.1)	0.453	5 (0.1)	37 (0.2)	0.401
Prostate	7 (0.2)	20 (0.1)	0.201	36 (0.9)	141 (0.7)	0.198
Thyroid	5 (0.1)	7 (0.0)	0.021 ^a^	23 (0.6)	93 (0.5)	0.372
Uterus	2 (0.1)	20 (0.1)	0.336	30 (0.8)	102 (0.5)	0.065
Melanoma	7 (0.2)	21 (0.1)	0.242	23 (0.6)	144 (0.7)	0.302
Stomach	1 (0.0)	9 (0.0)	0.568	7 (0.2)	55 (0.3)	0.250
Another site	14 (0.4)	17 (0.1)	<0.001 ^a^	21 (0.5)	98 (0.5)	0.791
Unknown primary	33 (0.8)	26 (0.1)	<0.001 ^a^	82 (2.1)	190 (1.0)	<0.001 ^a^

**^a^** statistically significant at a level of *p* < 0.05 after accounting for multiple comparisons using the Benjamini-Hochberg method. Comparison of malignancy rates in sarcoidosis patients and controls within 1 year before and after sarcoidosis diagnosis/index date versus more than 1 year. The table presents malignancy rates as the number of cases (*n*) and percentage (%) within each group. Statistical significance of comparisons between sarcoidosis patients and controls is indicated by *p*-values, with significant values (*p* < 0.05). Malignancies are categorized into “All Malignancies” “Hematologic” and “Solid” with further breakdowns for specific types and sites.

**Table 4:** Multivariate logistic regression analysis comparing rates of different malignancies between sarcoidosis and controls; overall, within 1 year period of diagnosis of sarcoidosis/index date, and over 1 year period.

Malignancy Site	Events (%) in Sarcoidosis Patients (*n* = 3993)	Events (%) in Controls (*n* = 19,856)	Adjusted Odds Ratio	95% Confidence Interval	*p*-Value
Overall					
All malignancies	779 (19.5)	2696 (13.6)	1.61	1.47 to 1.76	<0.001
Hematologic malignancies	168 (4.2)	292 (1.5)	2.94	2.41 to 3.57	<0.001
Solid malignancies	646 (16.2)	2474 (12.5)	1.41	1.27 to 1.55	<0.001
Lymphoma	133 (3.3)	184 (0.9)	3.67	2.92 to 4.63	<0.001
Leukemia	29 (0.7)	74 (0.4)	1.97	1.28 to 3.04	0.002
Breast	157 (3.9)	718 (3.6)	1.13	0.94 to 1.35	0.192
Melanoma	30 (0.8)	165 (0.8)	0.92	0.61 to 1.36	0.664
Lung	50 (1.3)	163 (0.8)	1.59	1.15 to 2.19	0.005
Sarcoma and soft tissue	25 (0.6)	41 (0.2)	2.71	1.61 to 4.58	<0.001
Gastrointestinal	113 (2.8)	526 (2.6)	1.08	0.88 to 1.34	0.456
Genitourinary	152 (3.8)	585 (2.9)	1.32	1.10 to 1.59	0.003
ENT	43 (1.1)	176 (0.9)	1.21	0.86 to 1.69	0.271
Within 1 year of sarcoidosis diagnosis/index date					
All malignancies	218 (5.5)	379 (1.9)	3.04	2.56 to 3.61	<0.001
Hematologic malignancies	72 (1.8)	33 (0.2)	11.37	7.43 to 17.4	<0.001
Solid malignancies	150 (3.8)	349 (1.8)	2.24	1.83 to 2.72	<0.001
Lymphoma	58 (1.5)	21 (0.1)	14.88	8.83 to 25.1	<0.001
Leukemia	9 (0.2)	9 (0.0)	4.94	1.95 to 12.50	0.001
Breast	15 (0.4)	87 (0.4)	0.90	0.52 to 1.55	0.696
Melanoma	7 (0.2)	21 (0.1)	1.66	0.70 to 3.91	0.250
Lung	20 (0.5)	22 (0.1)	4.49	2.44 to 8.26	<0.001
Sarcoma and soft tissue	7 (0.2)	8 (0.0)	3.42	1.18 to 9.91	0.024
Gastrointestinal	17 (0.4)	75 (0.4)	1.19	0.70 to 2.02	0.528
Genitourinary	33 (0.8)	85 (0.4)	1.93	1.29 to 2.90	0.001
ENT	9 (0.2)	23 (0.1)	2.05	0.94 to 4.44	0.069
Over 1 year of sarcoidosis diagnosis/index date					
All malignancies	561 (14.0)	2317 (11.7)	1.26	1.14 to 1.40	<0.001
Hematologic malignancies	96 (2.4)	259 (1.3)	1.83	1.44 to 2.33	<0.001
Solid malignancies	496 (12.4)	2125 (10.7)	1.21	1.09 to 1.35	<0.001
Lymphoma	75 (1.9)	165 (0.8)	2.23	1.68 to 2.96	<0.001
Leukemia	21 (0.5)	66 (0.3)	1.60	0.97 to 2.63	0.064
Breast	142 (3.6)	631 (3.2)	1.16	0.96 to 1.40	0.127
Melanoma	23 (0.6)	144 (0.7)	0.80	0.51 to 1.26	0.338
Lung	30 (0.8)	141 (0.7)	1.11	0.74 to 1.65	0.613
Sarcoma and soft tissue	18 (0.5)	33 (0.2)	2.52	1.38 to 4.61	0.003
Gastrointestinal	96 (2.4)	455 (2.3)	1.05	0.84 to 1.32	0.646
Genitourinary	123 (3.1)	507 (2.6)	1.23	1.00 to 1.50	0.047
ENT	34 (0.9)	153 (0.8)	1.09	0.75 to 1.58	0.665

Multivariate logistic regression analysis comparing malignancy rates between sarcoidosis patients and controls overall, within 1 year of sarcoidosis diagnosis/index date, and over 1 year post-diagnosis. Events in sarcoidosis patients and controls are presented as the number of cases (*n*) and percentage (%). Adjusted odds ratios (OR) with 95% confidence intervals (CI) are provided for each malignancy type, indicating the strength of association between sarcoidosis and specific malignancy risks. Significant *p*-values (*p* < 0.05) are noted to highlight statistically meaningful findings.

## Data Availability

The data used in this study are not available upon request due to the privacy policy of CHS.
